# Chitosan oligosaccharide improves intestinal homeostasis to achieve the protection for the epithelial barrier of female *Drosophila melanogaster* via regulating intestinal microflora

**DOI:** 10.1128/spectrum.03639-23

**Published:** 2024-02-27

**Authors:** Lu Wang, Cheng Zhang, Shuhang Fan, Jianfeng Wang, Weihao Zhou, Zhaohui Zhou, Yuhang Liu, Qianna Wang, Wei Liu, Xianjun Dai

**Affiliations:** 1 College of Life Sciences, China Jiliang University, Hangzhou, Zhejiang, China; 2 Hangzhou Original Seed Farm, Hangzhou, China; 3 Zhejiang Academy of Agricultural Sciences, Hangzhou, Zhejiang, China; 4 Key Laboratory of Specialty Agri-Product Quality and Hazard Controlling Technology of Zhejiang Province, Hangzhou, Zhejiang, China; Central Texas Veterans Health Care System, USA

**Keywords:** chitosan oligosaccharide, *Drosophila melanogaster*, oxidant stress, intestinal homeostasis, intestinal microflora

## Abstract

Chitosan oligosaccharide (COS) is a new type of marine functional oligosaccharide with biological activities such as regulating intestinal microflora and improving intestinal immunity. In this study, female *Drosophila melanogaster* was used as a model organism to evaluate the effect of COS on intestinal injury by H_2_O_2_ induction, and its mechanism was explored through the analysis of intestinal homeostasis. The results showed that 0.25% of COS could effectively prolong the lifespan of stressed female *D. melanogaster* by increasing its antioxidant capacity and maintaining intestinal homeostasis, which included protecting the mechanical barrier, promoting the chemical barrier, and regulating the biological barrier by affecting its autophagy and the antioxidant signaling pathway. Additionally, the protective effect of COS on the intestinal barrier and homeostasis of *D. melanogaster* under oxidative stress status is directly related to its regulation of the intestinal microflora, which could decrease excessive autophagy and activate the antioxidant system to promote health.

**IMPORTANCE:**

The epithelial barrier plays an important role in the organism’s health. Chitosan oligosaccharide (COS), a new potential prebiotic, exhibits excellent antioxidant capacity and anti-inflammatory effects. Our study elucidated the protective mechanisms of COS on the intestinal barrier of *Drosophila melanogaster* under oxidative stress, which could provide new insights into COS application in various industries, such as food, agriculture, and medicine.

## INTRODUCTION

Intestinal health relies on the establishment of homeostasis, a dynamic state formed by the interaction of the hosts (ZO-1, occludin, enterocytes, intestinal mucosa, and immune barrier), the intestinal environment (gut microbiota), nutrients, and metabolites, which are more pronounced in dietary habits (1). The integrity of the epithelial barrier and intestinal microbiota has been demonstrated to play important roles in intestinal homeostasis and the pathogenesis of certain diseases (2). When the body ingests toxic compounds, the number of dead intestinal epithelial cells increases, and intestinal stem cells proliferate excessively (3), which causes an imbalance in intestinal homeostasis. An imbalance between proliferative homeostasis and regenerative capacity is a hallmark of aging and age-related diseases (4).

To maintain long-term homeostasis in the barrier epithelia, gut immune function must be balanced with microbiota (5). Maintaining a healthy commensal population by preserving innate immune homeostasis in such epithelial cells is a promising approach to promoting health and longevity (6, 7). However, a reasonable composition of intestinal microorganisms can promote the integrity of intestinal barrier function and reduce the disordered proliferation of intestinal stem cells (8). Therefore, dietary ingredients could regulate intestinal homeostasis via the microflora affecting the intestinal barrier.

Chitosan oligosaccharide (COS), which is obtained by physical, enzymatic, or chemical hydrolysis (9, 10), is a derivative of chitosan composed of glucosamine linked by the β-(1→4)-glycosidic bonds and has shorter chain lengths, with less than 20% degree of polymerization (DP) (11) and physicochemical properties, including low viscosity, high water solubility (12), and nontoxicity (13). Due to the properties of COS, it has been regarded as a new potential prebiotic that exhibits excellent regulatory effects on intestinal bacteria and has been applied in various industries, such as food, agriculture, and medicine (14). For instance, COS shows antioxidant capacity by nourishing beneficial bacteria such as *Lactobacillus* and *Lactococcus* (15), and its anti-inflammatory effect is related to the enrichment of *Akkermansia*, which promotes the repair of inflammatory regions in mice (16). Additionally, COS treatment reduces the population of the bacterial community as a whole and increases the production of acetic acid. One *in vivo* study demonstrated that in mice, COS treatment promoted the population of Bacteroidetes but inhibited the Proteobacteria phylum. In the diabetic db/db mouse model, COS relieved gut dysbiosis by promoting *Akkermansia* and suppressing *Helicobacter* (17). However, the effect of COS on gut homeostasis remains unknown.

With its genetic amenability, strong conservation of cellular signaling pathways that regulate immune responses, and relatively simple composition of enteric microbes, the intestine of the *Drosophila melanogaster* is an ideal model system for investigating intestinal homeostasis (18). In this experiment, COS was fed on female *D. melanogaster* after the H_2_O_2_ challenge to evaluate the physiological status including the lifespan and structural and chemical immune homeostasis. The composition of intestinal microflora was examined to evaluate microorganism homeostasis for the H_2_O_2_-induced *D. melanogaster*. Finally, RNAi, sterile, and gnotobiotic stressful *D. melanogaster* were cultured to explore their relative mechanisms.

## RESULTS

### The effects of COS on the lifespan and antioxidant capacity of normal *D. melanogaster* under oxidative stress status

**Fig 1:**
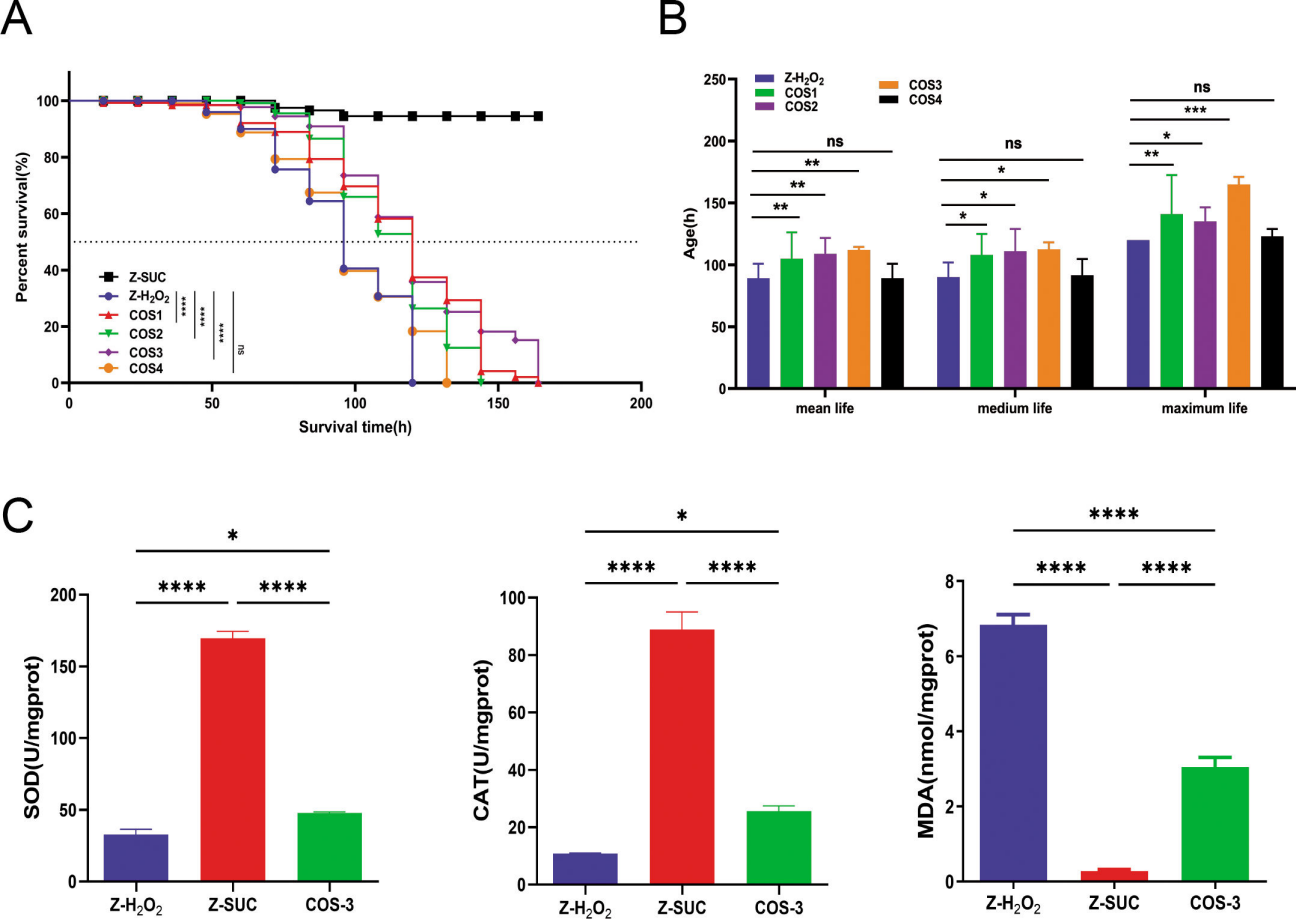
The effect of COS on the lifespan and antioxidant capacity of female *D. melanogaster* induced by H_2_O_2_. (**A, B**) Survival curve and mean lifespan, median lifespan, and maximum lifespan of female flies from the Z-SUC, Z-H_2_O_2_, 0.0625%, 0.125%, 0.25%, and 0.5% COS groups. (**C**) SOD and CAT activities and the MDA content of female flies from Z-H_2_O_2_, Z-SUC, and COS-3 (0.25%) groups. The results are expressed as the mean ± SEM (*n* = 3), and ns, *, **, ***, and **** indicate *P* > 0.05, *P* < 0.05, *P* < 0.01, *P* < 0.001, and *P* < 0.0001, respectively.

After 84 h of H_2_O_2_ treatment, the survival rate of female fruit flies in the Z-H_2_O_2_ group decreased to 56.25% (Fig. 1A), whereas those fed 0.0625%, 0.125%, 0.25%, and 0.5% COS were 68.75%, 82.50%, 86.25%, and 57.50%, respectively. Compared with the Z-H_2_O_2_ group, the mean lifespan of female flies fed COS increased by 17.68% (*P* < 0.01), 22.22% (*P* < 0.01), 25.76% (*P* < 0.01), and 0.38% (*P* > 0.05); the median lifespan significantly increased by 20.00% (*P* < 0.05), 23.33% (*P* < 0.05), 25.00% (*P* < 0.05), and 1.67% (*P* > 0.05); and the maximum lifespan increased by 17.50% (*P* < 0.01), 12.50% (*P* < 0.05), 37.50% (*P* < 0.001), and 2.50% (*P* > 0.05) (Fig. 1B). Therefore, 0.25% COS in the medium (COS-3) group could effectively alleviate the induced injury in female *D. melanogaster* by H_2_O_2_ and was used as the experimental group in the subsequent study. Compared with the Z-H_2_O_2_ group, the superoxide dismutase (SOD) activity in the tissues of female *D. melanogaster* fed 0.25% COS increased by 46.42% (*P* < 0.05), the catalase (CAT) activity increased by 136.41% (*P* < 0.05), and the malondialdehyde (MDA) content decreased by 55.37% (*P* < 0.0001) (Fig. 1C).

### The effects of COS on the intestinal mechanical barrier of structural homeostasis of female *D. melanogaster* under oxidative stress status

**Fig 2:**
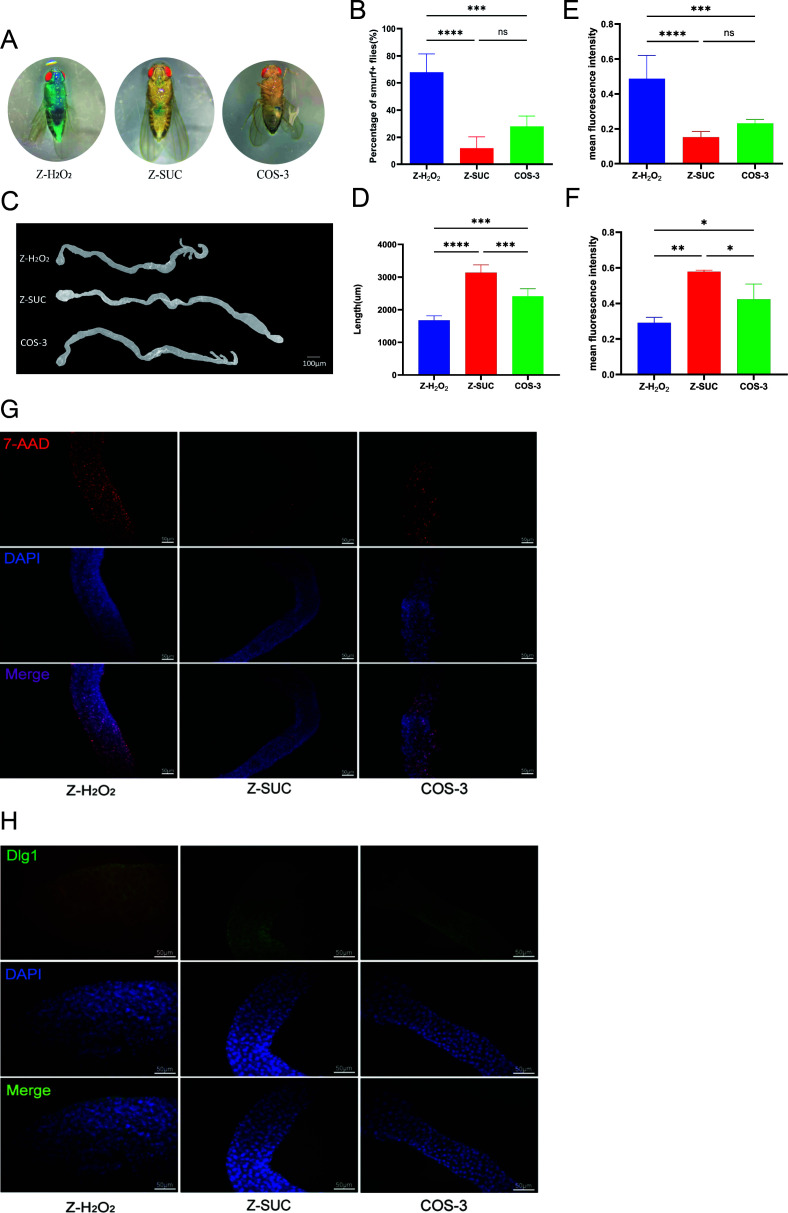
The effect of COS on intestinal structural homeostasis in *D. melanogaster* induced by H_2_O_2_. (**A, B**) Smurf staining and percentage of intestinal leakage. (**C, D**) The shape diagram and length of the intestine. (**E, G**) The fluorescence density and brightness of dead cells on the intestinal epithelium stained by 7-ADD. (**F, H**) The fluorescence intensity and brightness of dlg1 protein between the intestinal cells stained by anti-Dlg1 protein. The results are expressed as the mean ± SEM (*n* = 3), and *, **, ***, and **** indicate *P* > 0.05, *P* < 0.05, *P* < 0.01, *P* < 0.001, and *P* < 0.0001, respectively.

The Smurf test showed the lower intestinal leakage of *D. melanogaster* in the Z-SUC group (Fig. 2A), but compared with the Z-H_2_O_2_ group, the leakage rate of the COS-3 group showed a 58.8% (*P* < 0.001) reduction in Smurf flies according to the average 12%, 28%, and 68% leakage rate of Z-SUC, Z-H_2_O_2_, and COS-3 group, respectively (Fig. 2B). The intestinal tract of healthy fruit flies was long and symmetrical in the Z-SUC group (Fig. 2C), and the average intestinal length of *D. melanogaster* in the Z-SUC group was 3135 µm and that in the COS-3 group was 2415 µm, which was prolonged by 44.1% (*P* < 0.001) in contrast to the 1677-µm intestinal length in the Z-H_2_O_2_ group (Fig. 2D). The fluorescence density showed that, compared with the Z-H_2_O_2_ group, COS significantly reduced the number of dead intestinal epithelial cells stained by 7-ADD with 52.50% (*P* < 0.01) in Fig. 2E, and the difference could be found in Fig. 2G according to the red fluorescent brightness between the two groups. Compared with that in the Z-H_2_O_2_ group, the dlg1 protein content in the COS-3 group was significantly increased by 45.45% (*P* < 0.05) in Fig. 2F with the difference of green fluorescent brightness of the two groups (Fig. 2H). These results indicated that COS plays an important role in protecting the intestinal mechanical barrier.

### The regulating effects of COS on the intestinal stem cell (ISC) proliferation and differentiation of structural homeostasis of female *D. melanogaster* under oxidative stress status

**Fig 3:**
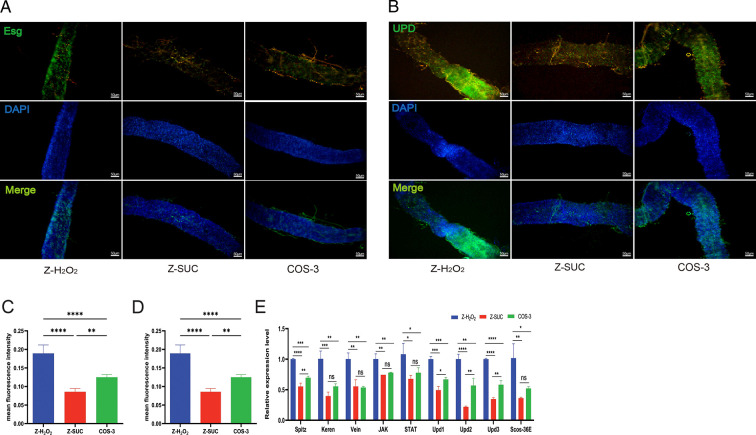
The effect of COS on stem cell homeostasis in female *D. melanogaster* induced by H_2_O_2_. (**A, B**) Green fluorescence images of precursor cells and intestinal cells, which respectively contained the GFP genes in the genome. (**C, D**) Average green fluorescence density of precursor cells and intestinal cells. (**E**) The expression levels of ligand genes in the EGFR and JAK/STAT signaling pathway. The results are expressed as the mean ± SEM (*n* = 3), and *, **, ***, and **** indicate *P* > 0.05, *P* < 0.05, *P* < 0.01, *P* < 0.001, and *P* < 0.0001, respectively.

The esg-Gal4;UAS-GFP and upd-Gal4;UAS-GFP *D. melanogaster,* respectively, carried green fluorescent protein (GFP) reporter genes in the genome of precursor cells and intestinal cells including enteroblast (EB) and enterocyte (EC) cells. The number of GFP-positive cells, precursor cells, in the COS-3 group was significantly lower than that in the Z-H_2_O_2_ group by 34.1% (*P* < 0.001) in Fig. 3C according to the green fluorescent brightness of the two groups (Fig. 3A). The number of GFP-positive cells, intestinal cells in the COS-3 group, was significantly lower than those in the Z-H_2_O_2_ group by 23.9% (*P* < 0.001) in Fig. 3D according to the green fluorescent brightness of the two groups (Fig. 3B). Additionally, compared with the Z-H_2_O_2_ group, the gene expression levels of Spitz, Keren, and Vein ligands of epidermal growth factor receptor (EGFR) signaling pathway in the COS-3 group significantly decreased by 30.32% (*P* < 0.001), 44.69% (*P* < 0.01), and 46.49% (*P* < 0.01), respectively, and those of JAK and STAT in the JAK/STAT signaling pathway and its ligands Upd1, Upd2, and Upd3 were significantly decreased by 22.31% (*P* < 0.01), 28.06% (*P* < 0.05), 33.13% (*P* < 0.001), 43.34% (*P* < 0.01), and 41.87% (*P* < 0.001) in the COS-3 group, respectively (Fig. 3E). Compared with the Z-H_2_O_2_ group, the transcription level of the target gene, Scos-36E, of the JAK/STAT signaling pathway also decreased by 48.75% (*P* < 0.05). The addition of COS can reduce the expression of related genes in the EGFR and JAK/STAT signaling pathways, which can decrease the proliferation and differentiation of intestinal stem cells.

### The effects of COS on intestinal chemical immune homeostasis of female *D. melanogaster* under oxidative stress status

**Fig 4:**
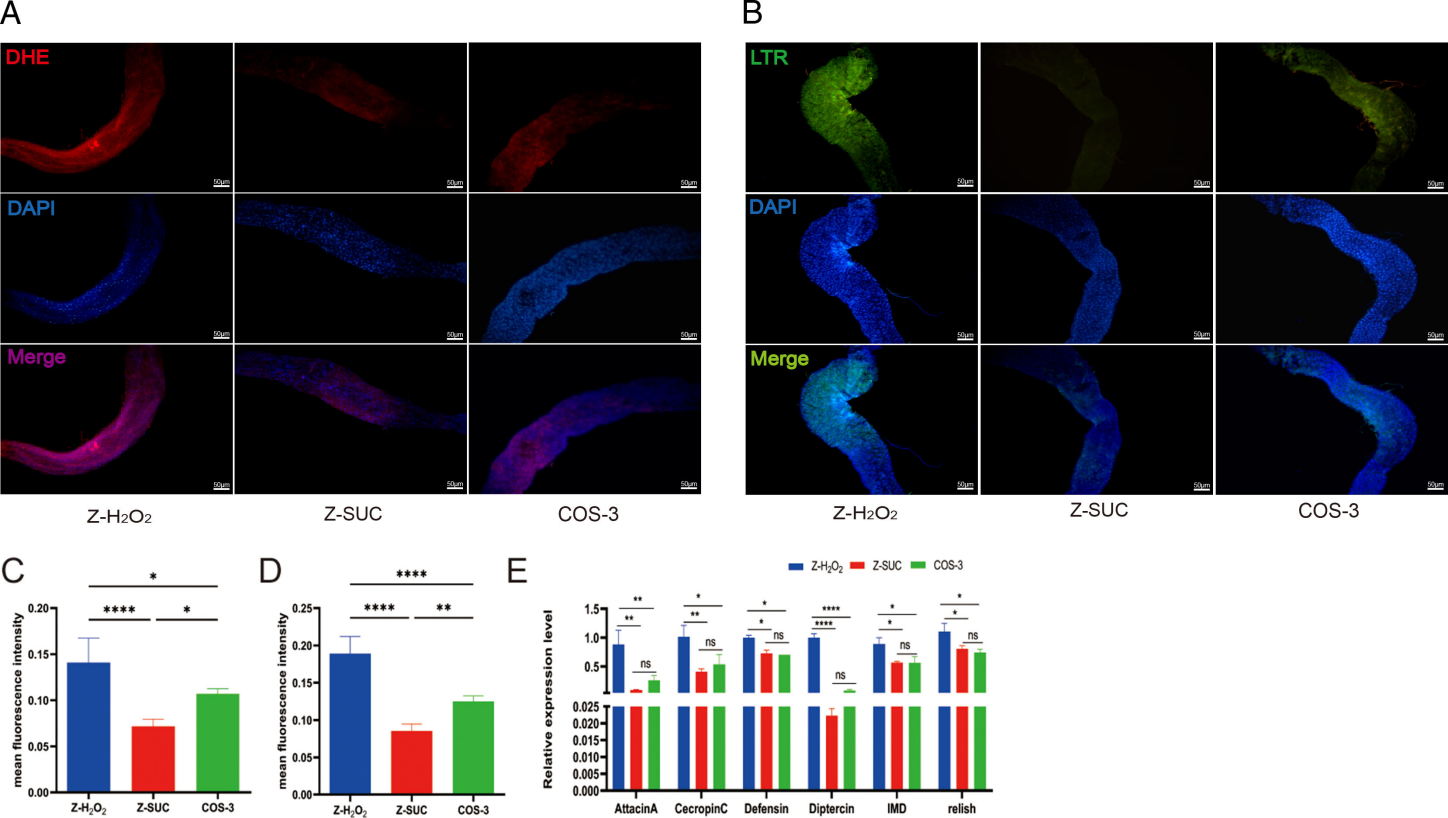
The effect of COS on ROS and lysosome content in the intestinal cells and gene expression of the IMD signaling pathway of female *D. melanogaster* induced by H_2_O_2_. (**A, C**) DHE staining and average fluorescence density of ROS in the intestinal cells. (**B, D**) LysoTracker Red staining and average fluorescence density of lysosomes in the intestinal cells. (**E**) The gene expression level of the IMD signaling pathway. The results are expressed as the mean ± SEM (*n* = 3), and *, **, ***, and **** indicate *P* > 0.05, *P* < 0.05, *P* < 0.01, *P* < 0.001, and *P* < 0.0001, respectively.

Compared with the Z-H_2_O_2_ group, the level of reactive oxygen species (ROS) in *D. melanogaster* fed COS decreased by 56.9% (*P* < 0.01) in Fig. 4A, and there is a significant difference in Fig. 4C according to the red fluorescent brightness of the two groups with dihydroethidium (DHE) staining, which indicated that COS has a positive effect on scavenging excess ROS caused by oxidative stress. In the Z-SUC group, there were fewer lysosomes in the intestinal tract of *D. melanogaster* based on the green fluorescent brightness in Fig. 4B, and the height of the column in Fig. 4D, but the lysosomal content in the intestinal cells increased after the stimulation with H_2_O_2_ in the Z-H_2_O_2_ group (Fig. 4B). Compared with the Z-H_2_O_2_ group, the average fluorescence density of lysosomes in the COS-3 group decreased by 56.34% (*P* < 0.0001) in Fig. 4D, indicating that COS can effectively alleviate H_2_O_2_-induced intestinal damage in female *D. melanogaster*. H_2_O_2_ treatment significantly increased the expression of the intestinal immune deficiency (IMD) signaling pathway (Fig. 4E). Compared with the Z-H_2_O_2_ group, the expression of the *Attacin A*, *Cecropin C*, *Defensin*, *Diptercin*, IMD, and Relish genes significantly decreased by 70.11% (*P* < 0.01), 46.79% (*P* < 0.05), 29.52% (*P* < 0.05), 91.81% (*P* < 0.0001), 36.78% (*P* < 0.05), and 32.72% (*P* < 0.05), respectively, in the COS-3 group (Fig. 4E), indicating that COS could reduce the expression of antibacterial peptide genes in *D. melanogaster* induced by H_2_O_2_.

### The regulation of COS on the intestinal microflora of female *D. melanogaster* under oxidative stress status

**Fig 5:**
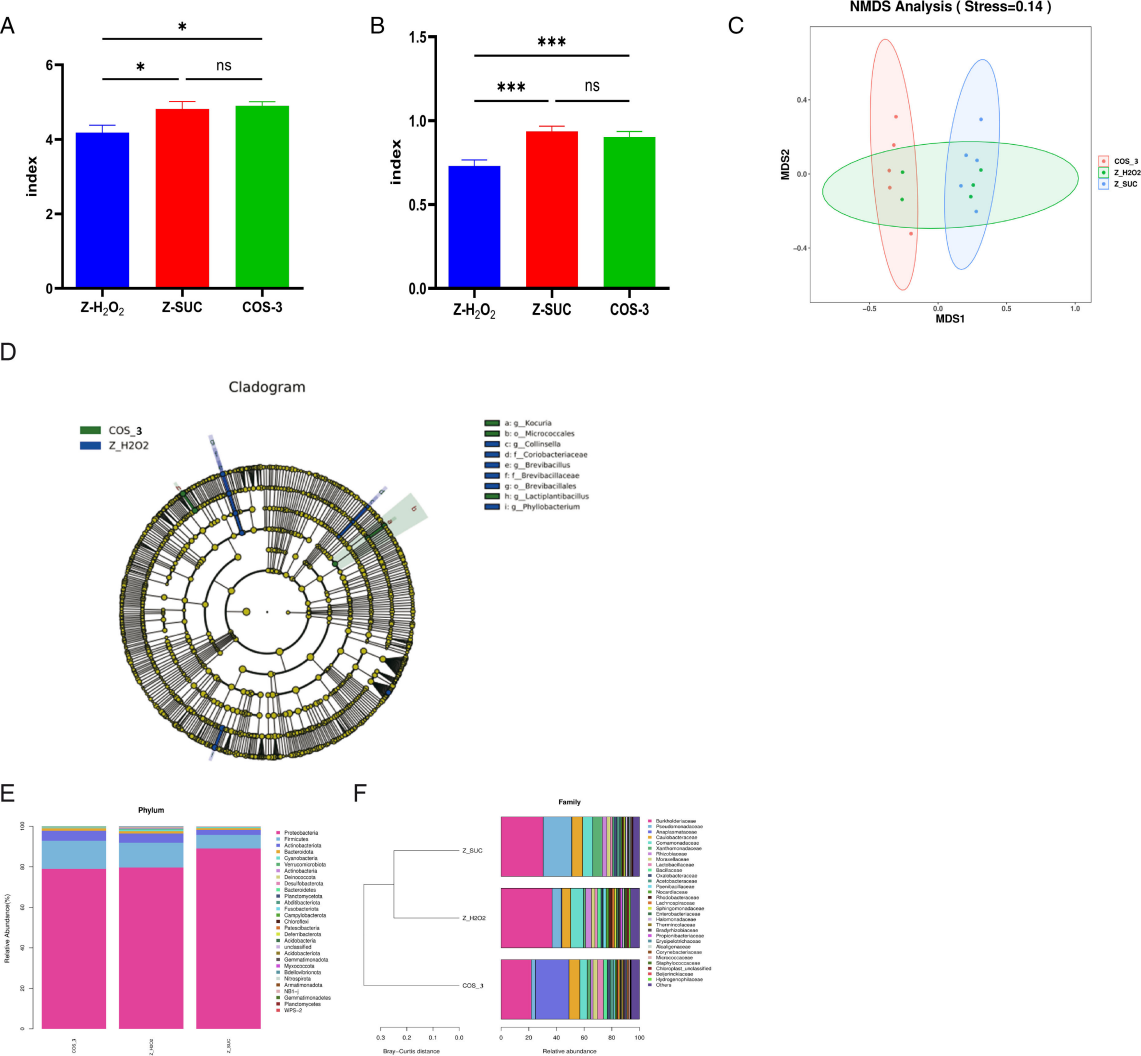
The effect of COS on the intestinal microflora in female *D. melanogaster* induced by H_2_O_2_. (**A, B, C**) Shannon and Simpson indices, and the NMDS analysis. (**D**) LEfSe diagram. (**E, F**) The phylum and family levels. The results are expressed as the mean ± SEM (*n* = 3), and ns, * and *** indicate *P* > 0.05, *P* < 0.05, and *P* < 0.001, respectively.

According to the α-diversity analysis, the Shannon (Fig. 5A) and Simpson indices (Fig. 5B) in the COS-3 group increased by 17.14% (*P* < 0.05) and 23.65% (*P* < 0.001), respectively, compared with those in the Z-H_2_O_2_ group. β-Diversity analysis using non-metric multidimensional scaling (NMDS) showed that there were separated regions between the Z-SUC and COS-3 groups (Fig. 5C), and the Z-H_2_O_2_ group had a wider distribution range of intestinal microorganisms, which indicated that COS could significantly change the distribution pattern of intestinal microflora.

According to the linear discriminant analysis effect size (LEfSe) analysis (Fig. 5D), the dominant microorganisms in the COS-3 group included *Cocuria*, *Micrococcales*, and *Lactobacillus plantarum*, and the dominant microorganisms of the Z-H_2_O_2_ group included *Collins*, *Coriobacteriaceae*, *Brevibacillus,* and *Phyllobacterium*. Compared with those in the Z-H_2_O_2_ group, the intake of COS decreased the relative abundance of Proteobacteria from 83.07% to 72.57% (*P* < 0.05), respectively, and increased the abundance of Firmicutes and Bacteroides from 9.30% to 13.06% (*P* < 0.05) and 1.13% to 1.37% (*P* > 0.05) at the phylum level (Fig. 5E). At the family level (Fig. 5F), *Burkholderia*, *Pseudomonas*, *Anaplamataceae*, *Caulobacteraceae*, *Comamonia,* and *Lactobacilliaceae* dominated the system, and the abundance of *Lactobacilliaceae* had a significant change with COS supplement. Compared with the Z-H_2_O_2_ group, *Lactobacilliaceae* abundance increased by 166.07% (*P* < 0.01) in the COS-3 group. The above results indicated that the COS intake could significantly regulate the intestinal microflora and promote the colonization of beneficial bacteria.

### The role of intestinal microflora in alleviating intestinal injury in female *D. melanogaster* under oxidative stress status

**Fig 6:**
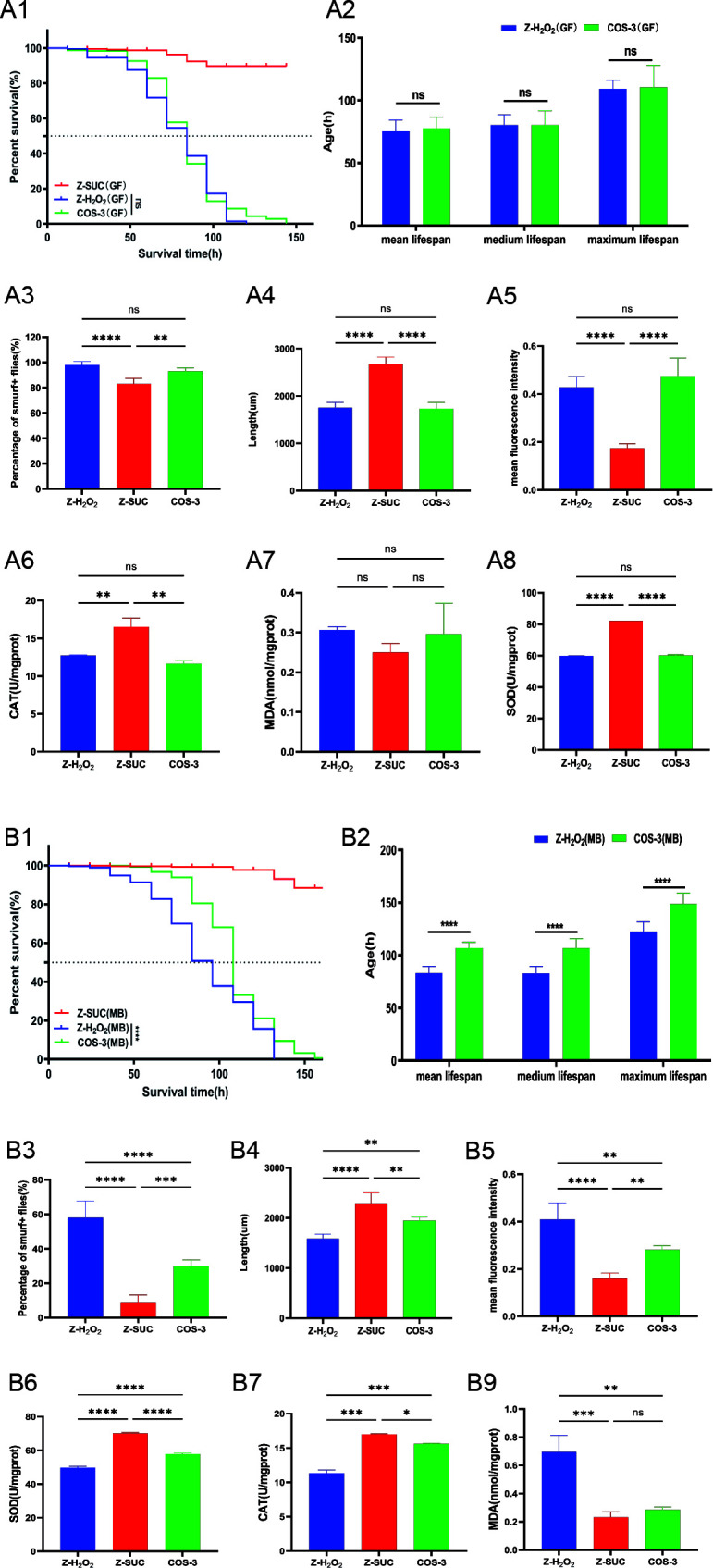
The effect of COS on the lifespan, intestinal structure, and oxidation level of sterile (A) and gnotobiotic (B) *D. melanogaster*. (**A1, B1**) Survival curve. (**A2, B2**) Mean, median, and maximum lifespan. (**A3, B3**)The percentage of intestinal leakage with Smurf trial. (**A4, B4**) Intestinal length. (**A5, B5**) The fluorescence density of dead cells on the intestinal epithelium. (**A6, B6**) SOD activity. (**A7, B7**) CAT activity. (**A8, B8**) MDA content. The results are expressed as the mean ± SEM (*n* = 3), and ns, *, **, ***, and **** indicate *P* > 0.05, *P* < 0.05, *P* < 0.01, *P* < 0.001, and *P* < 0.0001, respectively.

The 16S rRNA sequencing analysis showed that dietary supplementation with COS increased the intestinal microbial diversity of female flies. However, there were no significant differences in the survival curve (Fig. 6A1), lifespan (Fig. 6A2), intestinal leakage (Fig. 6A3), length (Fig. 6A4), epithelial cell mortality (Fig. 6A5), SOD activity, (Fig. 6A6), CAT activity (Fig. 6A7), or MDA content (Fig. 6A8) of sterile *D. melanogaster* between the Z-H_2_O_2_ and COS-3 groups. Additionally, according to the gnotobiotic *D. melanogaster* experiment, there were significant differences in the survival curve (Fig. 6B1), lifespan (Fig. 6B2), intestinal leakage (Fig. 6B3), length (Fig. 6B4), epithelial cell mortality (Fig. 6B5), SOD activity (Fig. 6B6), CAT activity (Fig. 6B7), and MDA content (Fig. 6B8) between the Z-H_2_O_2_ and COS-3 groups. Therefore, it is suggested that the intestinal microflora plays an important role in protecting the intestines of female flies induced by H_2_O_2_.

### The effect of COS on the intestinal signaling pathway of female *D. melanogaster* under oxidative stress status

**Fig 7:**
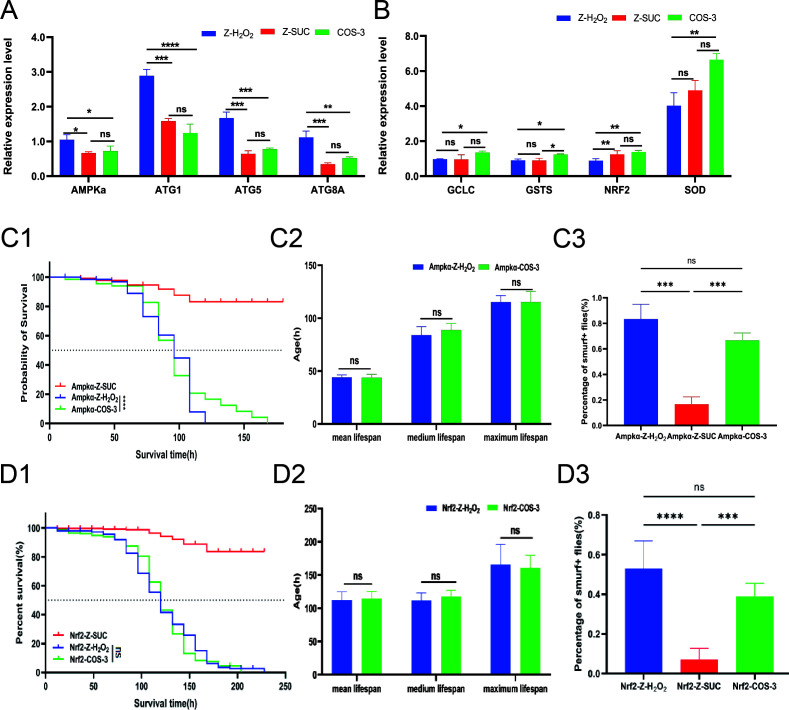
The effect of COS on the gene expression of the intestinal signaling pathway of induced female *D. melanogaster,* and physiological performance of female AMPKɑ and Nrf2-RNAi *D. melanogaster* induced by H_2_O_2_. (**A, B**) The gene expression of autophagy and antioxidant signaling pathway in the regular female flies. (**C1, D1**) Survival curve of female AMPKɑ and Nrf2-RNAi flies. (**C2, D2**) The lifespan of female AMPKɑ and Nrf2-RNAi flies. (**C3, D3**) Percentage of intestinal leakage with Smurf^+^ trial for female AMPKɑ and Nrf2-RNAi flies. The results are expressed as the mean ± SEM (*n* = 3), and ns, *, **, ***, and **** indicate *P* > 0.05, *P* < 0.05, *P* < 0.01, *P* < 0.001, and *P* < 0.0001, respectively.

Compared with those of the Z-H_2_O_2_ group, the expression levels of the AMPKα, Atg1, Atg5, and Atg8a genes were significantly downregulated by 31.12% (*P* < 0.05), 56.85% (*P* < 0.0001), 53.42% (*P* < 0.001), and 53.44% (*P* < 0.01), respectively, in female *D. melanogaster* treated with H_2_O_2_ in the COS-3 group (Fig. 7A), which indicated that COS could alleviate excessive autophagy in the intestine. Compared with those in the Z-H_2_O_2_ group, the expression levels of the GCL, GSTS, Nrf2, and SOD genes in the COS-3 group were increased by 39.51% (*P* < 0.05), 36.01% (*P* < 0.05), 55.65% (*P* < 0.01), and 65.13% (*P* < 0.01), respectively (Fig. 7B). There was no significant difference in the survival curve (Fig. 7C1 and D1), lifespan (Fig. 7C2 and D2), and intestinal leakage (Fig. 7C3 and D3) of AMPKɑ/Nrf2-RNAi flies between the Z-H_2_O_2_ and COS-3 groups.

## DISCUSSION

The intestinal barrier can be divided into mechanical, chemical, and biological components that form the basis of intestinal homeostasis. The mechanical barrier consists of intact intestinal mucosal epithelial cells and tight junction proteins, which can prevent the invasion of bacteria and macromolecules (19), and the homeostasis of mucosal epithelial cells is based on the delicate regulation of epithelial proliferation and differentiation. The chemical immune barrier refers to the intestinal fluid, associated proteases, and large amounts of organic acids produced by diverse gut microbiota (20) and includes ROS, AMP, and lysosomes in epithelial cells. The biological barrier consists of anaerobic bacteria in the intestinal tract that can withstand colonization by both pathogenic and exogenous bacteria, collectively referred to as gut microbiota (21). In the experiment, when the female *D. melanogaster* suffered from oxidative stress *via* the digestive tract, its lifespan was significantly affected, in contrast to that in the Z-SUC group. A certain concentration of COS could significantly extend the lifespan of female *D. melanogaster* in oxidative stress status with improving antioxidant capacity (Fig. 1). However, it is necessary to elucidate how COS ameliorates intestinal stress because H_2_O_2_ induction can seriously damage the intestine.

First, COS had a significant protective effect on the mechanical barrier of the intestine in *D. melanogaster* treated with H_2_O_2_. COS reduced the intestinal leakage of *D. melanogaster* under oxidative stress and increased the length of the damaged intestinal tract (Fig. 2B and D), indicating that intestinal injury under the action of COS was relatively light, which was reflected in the mortality of intestinal cells and the increased content of dlg1 protein among intestinal cells (Fig. 2E and F), while the intestinal barrier was maintained at the level of cell connection, including adhesive connection and spacer connection, which had been tested and consistent with the reported conclusion that the loss of dlg1 protein in *D. melanogaster* represents the failure of barrier function (22). A more complete intestinal barrier could have an intimate relationship with the proliferation and differentiation homeostasis of intestinal ISCs. According to a previous report, the oxidative burst induced by the host consecutive to Ecc15 infection is a major inducer of ISC activation, and infection with this bacterium activates both the JAK-STAT and c-Jun NH2 terminal kinase (JNK) pathways in ISCs to promote proliferation (23). Additionally, the excessive generation of ROS with aging may contribute to the excessive proliferation and differentiation of intestinal stem cells, thereby impairing gut homeostasis and affecting the lifespan (24). In this experiment, the addition of COS reduced the expression of genes related to the EGFR and JAK/STAT signaling pathways (Fig. 3E), which could decrease the activity of intestinal stem cells (Fig. 3C). At the same time, the number of precursor cells and enteroblast cells progressively showed a low level of stem cell proliferation and differentiation in the intestine of *D. melanogaster* in the COS-3 group (Fig. 3D), which promoted intestinal structural homeostasis to protect the intestinal mechanical barrier of *D. melanogaster* under oxidative stress. COS had a significant protective effect on the chemical barrier of the intestine in *D. melanogaster* treated with H_2_O_2_.

Second, studies have shown that innate immunity is crucial for maintaining intestinal homeostasis and is conserved between invertebrates and vertebrates (25, 26). COS reduced the expression of antimicrobial peptide (AMP)-related genes (Fig. 4E) and lysosome numbers (Fig. 4D) and increased the scavenging of excess ROS (Fig. 4C) in *D. melanogaster* induced by H_2_O_2_. AMPs, as well as ROS produced by dual oxidase (DUOX), are key substances in the innate immune system (27). In *D. melanogaster*, the Toll and IMD pathways are two major signaling pathways involved in innate immune responses (28), which could control the expression of AMPs (29). Usually, the Gram-negative bacterial infection results in the expression of another set of AMPs, such as *Attacin A*, *Cecropin C, Defensin*, and *Diptercin* through activating the IMD signaling pathway (30). Additionally, lysosomes are single membrane-bound organelles containing acid hydrolysates as the products of cell autophagy, which is a process that removes the damaged proteins and organelles from cells under oxidative stress and plays an important role in maintaining intestinal homeostasis (31). Therefore, the lysosome reduction suggests that COS can effectively alleviate H_2_O_2_-induced intestinal injury in female *D. melanogaster* by decreasing the autophagy intensity.

Finally, COS had a significant regulatory effect on the biological barrier of the intestine in *D. melanogaster* treated with H_2_O_2_. The intestinal microflora participates in host food digestion, metabolic adaptation, and immune system regulation (32). According to the 16S rDNA sequencing analysis, COS promoted the diversity of intestinal microflora (Fig. 5A and B) and concentrated them in relatively compact areas (Fig. 5C), indicating that COS could significantly change the distribution pattern of intestinal microflora. In the LEfSe diagram, the dominant flora of the COS-3 group was *Lactobacillus plantarum* (Fig. 5D), which can produce bacteriostatic substances, such as lactic acid, acetic acid, and AMPs (33, 34). The dominant bacteria in the Z-H_2_O_2_ group included the genus *Collins*, the family *Coriobacteriaceae*, the genus *Brevibacilli*, and the genus *Phyllobacterium* (Fig. 5D). Vandeputte et al. reported that the genus *Collins* is highly abundant in the intestines of constipated people (35). *Coriobacteriaceae* has been shown to be related to obesity-related metabolic parameters (33, 36). At the phylum level, COS can reduce the relative abundance of Proteobacteria while increasing the abundance of Firmicutes and Bacteroides (Fig. 5E). When the abundance of Proteus increases, the probability of intestinal microflora disorders could lead to inflammatory reactions (37). Firmicutes can decompose some dietary fibers, which play an important role in health (38, 39). At the same time, the increase in Firmicutes could promote the content of secondary bile acids, thereby playing an immunomodulatory role (40). Additionally, Bacteroides are anaerobic bacteria that can produce short-chain fatty acids that provide energy for intestinal repair, protect intestinal barriers, and inhibit inflammatory responses (41). At the family level, the abundance of *Lactobacilli* significantly increased after ingestion of COS (Fig. 5F), which is the most common indicator to determine whether the intestinal tract is healthy (42). The above results indicated that the intake of COS can increase the colonization of beneficial bacteria in the intestinal tract while reducing the harmful bacteria in the intestine of female *D. melanogaster* under oxidative stress to maintain intestinal homeostasis. Furthermore, sterile and gnotobiotic *D. melanogaster* exposed to oxidative stress verified that COS supplementation plays a key role by regulating the composition of intestinal microorganisms, affecting the intestinal structure and oxidative level (Fig. 6A and B).

Collectively, H_2_O_2_ can cause intestinal epithelial damage and increase cell death by stimulating the intestinal tract of *D. melanogaster*, which shortens the lifespan of fruit flies. However, promoting intestinal homeostasis and reducing epithelial barrier dysfunction contribute to lifespan extension (43, 44), and enhancing ROS scavenging improves intestinal homeostasis, which could promote body health and retard aging (45). Nrf2 is considered to be an important factor in maintaining the redox balance of cells (46), and it has been reported that H_2_O_2_-induced oxidative stress adaptation strongly depends on the increase in the 20S proteasome mediated by the Nrf2 transcription factor (47, 48). Therefore, the Keap1-Nrf2 signaling pathway can enhance their resistance to external stress (49). Additionally, the autophagy signaling pathway could maintain energy homeostasis by decomposing intracellular components (50). While excessive autophagy can also cause adverse effects on biological individuals, its balance is important for maintaining intestinal homeostasis (51). In the experiment, the gene expression levels of autophagy and Keap1-Nrf2 signaling pathway indicated a significant difference between the COS-3 and Z-H_2_O_2_ groups (Fig. 7A and B), which showed that COS could downregulate the autophagy signaling pathway and promote the Keap1-Nrf2 signaling pathway. These results may be due to the regulation of gut microbiota and activation of antioxidant pathways with COS supplement, which alleviates the damage caused by H_2_O_2_, while the reduction of damage could downregulate the expression of autophagy genes. Furthermore, AMPKα and Nrf2-RNAi *D. melanogaster* did not exhibit higher survival rate in the AMPKα and Nrf2-RNAi-COS-3 groups in contrast with those in the AMPKα and Nrf2-RNAi-Z-H_2_O_2_ groups (Fig. 7C and D). The above results have progressively proven that COS can activate the antioxidant defense system and repress autophagy signaling to maintain intestinal homeostasis.

In conclusion, the 0.25% COS in the medium significantly prolonged the lifespan of *D. melanogaster* by improving the intestinal structure, regulating chemical immunity, inhibiting the proliferation and differentiation of intestinal stem cells, and enhancing the activity of antioxidant enzymes in the body. The protective effect of COS on the intestinal barrier of *D. melanogaster* under oxidative stress is directly related to its regulation of the intestinal microflora, which could decrease excessive autophagy and activate the antioxidant system to promote intestinal homeostasis. These results provide new insights into the mechanisms through which prebiotics affect intestinal homeostasis.

## MATERIAL AND METHODS

### Oligosaccharide and chemicals

COS with deacetylation degrees greater than 95% came from Dalian Glycobio Co., Ltd. (Dalian, China). Glucosamine was the only monosaccharide, and two to eight DP oligomers (Mw ≈856 Da) comprised 33.6% disaccharides, 16.9% trisaccharides, 15.8% tetrasaccharides, 12.4% pentasaccharides, 8.3% hexasaccharides, 7.1% heptasaccharides, and 5.9% octasaccharides. Reagents for culture medium included sucrose, maize flour, AGAR powder, and yeast powder (Hangzhou Best Biological Technology Co., Ltd). Chemicals of analytical grade in the experiment included the 4',6-diamidino-2-phenylindole (DAPI, Beijing Solarbio Science & Technology Co., Ltd.), DHE and Lyso-Tracker Red (LTR, Shanghai 4A Biotech Co., Ltd), enoglaucine disodium salt (Sigma Co., Ltd, USA), anhydrous ethanol (Shanghai Cloud Chemical Co., Ltd.), propionate, and glacial acetic acid (Hangzhou Gaojing Fine Chemical Co., Ltd.). Antibodies included mouse anti-Dlg1 protein (4F3 Discs86 large, 1:200, Abcam) and anti-mouse Alexa Fluor-48887 (ab150113, Abcam). The antioxidant assay kits included SOD, CAT, and MDA (Nanjing Jiancheng Bioengineering Co., Ltd). The gene-detecting kits included TB Green Premix Ex Taq, RNAiso Plus, and PrimeScript RT with gDNA Eraser (Beijing Subsection of TAKARA BIO Inc.).

### *D. melanogaster* strains and maintenance

*D. melanogaster* of CS lines (*Drosophila* Stock Center, Shanghai Academy of Life Sciences, Chinese Academy of Sciences), NP3084-gal4 flies (Number: DGRC113094, *Drosophila* Genetics Resource Center), UAS-AMPK/Nrf2 (CncC)-RNAi flies (Number: THU5248/TH04336.N, Tsinghua University *Drosophila* Stocks Center), esg-Gal4;UAS-GFP/CyO (Number: TB00044, Fungene Biotechnology Co., Ltd.), and upd3-GAL4;UAS-GFP/CyO (Number: THJ0199, Fungene Biotechnology Co., Ltd.) flies were cultured in the environment of 25°C and 55% relative humidity with a 12 h light/12 h dark cycle. AMPKɑ/Nrf2-RNAi, sterile, and gnotobiotic flies were prepared according to our previous method (52).

### Female *D. melanogaster* model of oxidative stress and its lifespan and antioxidant capacity

The anesthetized flies (within 8 h of birth) were randomly divided into the Z-SUC and Z-H_2_O_2_ and COS-1, COS-2, COS-3, and COS-4 groups and cultured on basal diets supplemented with 0 and COS at different concentrations of 0.0625%, 0.125%, 0.25%, and 0.5% to produce experimental flies. Two hundred newborn flies, respectively, from the Z-SUC, Z-H_2_O_2_, and each COS group were collected into a tube with filter paper containing 5% SUC, 5% SUC +3% H_2_O_2_, and 5% SUC +3% H_2_O_2_ + 0.0625%, 0.125%, 0.25%, and 0.5% COS solution. The survival rate, lifespan, and antioxidant capacity were examined according to our previous method (52).

### The mechanical barrier assay of female *D. melanogaster* intestine

The intestinal length, permeability, and survival rate of intestinal cells were examined according to our previous method (53). Immunostaining for the dlg1 protein assay was implemented as described previously with some modifications (54). Intestinal samples fixed with 4% paraformaldehyde for 30 min were rinsed with PBS + 0.5% Triton (PBS-Tx) for 5 min three times. Blocking was performed with 3% BSA in PBS-Tx for 30 min. Primary antibody (mouse anti-dlg1 protein, the Developmental Studies Hybridoma Bank) was added 1:200 in 3% BSA in PBS-Tx and incubated at 4°C overnight. The intestinal samples were rinsed three times with PBS-Tx for 5 mins. For the secondary antibody incubation, anti-mouse AlexaFluor-488 (ab150113, Abcam) was added 1:4,000 in 3% BSA in PBS-Tx for 10 h at 4°C. Samples were rinsed five times with PBS-Tx for 5 min and were stained with DAPI (1 µg/ml) for 6–7 min at room temperature. The samples were washed four times with PBS-Tx for 3 min. The intestines were mounted and observed under a fluorescence microscope. ImageJ was used to quantify the dlg protein intensity. Intestinal stem cell proliferation and differentiation were detected in esg-Gal4;UAS-GFP/CyO and upd3-GAL4;UAS-GFP/CyO flies. The intestines were dissected and immediately fixed in 4% paraformaldehyde. The cells were then washed three times with PBS. The intestines were then washed with PBS three times, stained with 1 µg/ml DAPI, washed with PBS five times, and observed under a fluorescence microscope. ImageJ software was used to measure the fluorescence of the GFP^+^ cells.

### The chemical immune homeostasis of assay of female *D. melanogaster* intestine

The ROS were examined using our previous DHE method (53). LTR was used to achieve specific fluorescent labeling of the lysosomes. Briefly, the intestinal samples were fixed with 4% paraformaldehyde for 30 min. PBS solution was used to wash the samples three times. LTR (50 nM) needs to be preheated at 37°C for 10 min before use. After combining with LTR for 5 min, the intestines were washed three times with PBS. Next, the intestines were stained with DAPI (1 µg/ml) for 7–8 min, washed five times with PBS, and mounted with 70% glycerol. Finally, they were observed using a fluorescence microscope, and ImageJ software was used to quantify the fluorescence intensity.

### Determination of gene expression and 16S rDNA analysis of microorganisms in the intestine

Total RNA was prepared and reversely transcribed into the cDNA template, and RT-PCR and Primer sequences were performed according to our previous method (53). DNA samples were extracted, and the V3-V4 regions were amplified according to our previous method (53). The 16S rDNA sequencing of the amplicon pools was executed on a NovaSeq PE250 platform by Library Quantification Kit for Illumina (Kapa Biosciences, USA), and the size and quantity were assessed using an Agilent 2100 Bioanalyzer (Agilent, USA). QIIME2 and R language (v3.5.2) were, respectively, used to evaluate α- and β-diversity and draw the figures.

### Statistical analysis

The data statistics and images were, respectively, analyzed using GraphPad Prism 9.0 (GraphPad Software, San Diego, USA) and ImageJ (National Institutes of Health, Bethesda, USA). Differences were determined using the one-way analysis of variance and Tukey’s multiple comparison test. All data are indicated as mean ± SEM and based on three replicates in each group, and statistical significance was set at *P* < 0.05.

## Data Availability

All raw sequencing data had be submitted to China national genomic data center database (CNGDC) under the accession numbers subCRA005248 and subCRA005151.

## AFTER EPUB

*[This article was published on 27 February 2024 with errors in Fig. 7. The figure was corrected in the current version, posted on 2 April 2024.]*
