# Correction for Wang et al., “Chitosan oligosaccharide improves intestinal homeostasis to achieve the protection for the epithelial barrier of female *Drosophila melanogaster* via regulating intestinal microflora”

**DOI:** 10.1128/spectrum.01174-24

**Published:** 2024-06-26

**Authors:** Lu Wang, Cheng Zhang, Shuhang Fan, Jianfeng Wang, Weihao Zhou, Zhaohui Zhou, Yuhang Liu, Qianna Wang, Wei Liu, Xianjun Dai

## AUTHOR CORRECTION

Volume 12, no. 4, e03639-23, 2024, https://doi.org/10.1128/spectrum.03639-23. Page 14: The following should be added to Acknowledgments: “…and Project of Innovation and Entrepreneurship Training for National Undergraduates (202210356).”

